# Sinomenine Inhibits the Progression of Bladder Cancer Cells by Downregulating LncRNA-HEIH Expression

**DOI:** 10.1155/2021/4699529

**Published:** 2021-11-01

**Authors:** Haili Xu, Jiayan Dong, Junhui Hou, Rui Gao

**Affiliations:** ^1^Department of Oncology, Jinan Municipal Hospital of Traditional Chinese Medicine, Jinan 250012, China; ^2^Department of Tumor Radiotherapy, Zhangqiu District People's Hospital, Jinan 250200, China; ^3^Department of Tumor Radiotherapy (III), Affiliated Qingdao Center Hospital, Qingdao University, Qingdao 2266000, China

## Abstract

**Background:**

Sinomenine has been reported to effectively repress the progression of lung cancer and breast cancer. However, the effects of sinomenine in bladder cancer are not well understood. The purpose of this study was to evaluate the effects of sinomenine in bladder cancer.

**Methods:**

The mRNA expression of HEIH in bladder cancer cells was measured by RT-qPCR. T24 and SW780 cells were treated with sinomenine for 24 hours. Cell viability was detected by the MTT assay. Cell migration and invasion were detected by the transwell assay. Western blotting assay was performed to assess the protein expression of Bcl-2, Bax, and caspase-3.

**Results:**

Sinomenine significantly suppressed cell viability in T24 and SW780 cells. Moreover, cell migration and invasion were significantly inhibited by sinomenine. Sinomenine accelerated the expression of Bax and caspase-3 but decreased the expression of Bcl-2. HEIH was upregulated in bladder cancer cells compared with normal bladder epithelial cells. Besides this, we noticed that HEIH knockdown blocked cell proliferation, migration, and invasion but facilitated cell apoptosis in bladder cancer cells. Additionally, HEIH reversed the suppression of the progression induced by sinomenine.

**Conclusion:**

Sinomenine was observed to suppress cell progression of bladder cancer cells by inhibiting HEIH expression. Our findings suggested that the use of sinomenine might be an effective treatment for bladder cancer.

## 1. Introduction

Bladder cancer, the most common malignant tumor of the urinary system, is a malignant tumor that occurs in the bladder mucosa. Globally, there are approximately 570,000 new cases of bladder cancer and 210,000 deaths every year [1]. At present, the cause of bladder cancer is associated with many factors, such as smoking, diet, occupational exposure, long-term exposure to high-risk chemicals, and activation of related oncogenes [2]. The treatment of bladder cancer is mainly surgery and adjuvant chemotherapy. However, all available treatments have adverse effects, and the prognosis is often not ideal [3, 4]. Therefore, it is essential to search for effective remedies to reduce the mortality of bladder cancer.

Chinese medicinal herbs are medicine used in traditional Chinese medicine (TCM). In recent years, the benefits of Chinese medicinal herbs in cancer treatment have attracted the attention of scholars. It has been reported that TCM treatment can effectively reduce postoperative complications, control the metastasis and diffusion of cancer cells, promote postoperative recovery, and enhance immunity [5, 6]. Kaempferol was reported to repress the proliferation of bladder cancer cells by blocking the expression of cyclin D1, CDK4, p-Akt, Bid, Bcl-XL, and McL-1 and promoting the expressions of p53, p21, p38, p-ATM, Bax, Bid, and p-BRCA1 [7]. Almeida et al. observed that resveratrol reduced cell proliferation and caused DNA damage in bladder cancer cells [8].

Sinomenine is a monomer alkaloid component extracted from *Caulis Sinomenii*, which belongs to isoquinoline alkaloid. Sinomenine has anti-inflammatory, antirheumatism, antitumor, sedation, antihypertensive, antiarrhythmia, immunosuppression, and other pharmacological effects [9–11]. Numerous experiments have shown that sinomenine can inhibit the development of breast cancer, stomach cancer, lung cancer, and ovarian cancer [12–14]. Bai et al. reported that sinomenine restrained cell migration and proliferation by suppressing the expression of *α*7 nicotinic acetylcholine, TTF-1, SP-1, and p-ERK/ERK in lung cancer [15]. Moreover, sinomenine suppressed cell invasion and growth by inhibiting the PI3K/Akt/mTOR pathway in breast cancer side population cells [16]. At the same time, the role of sinomenine on the development of bladder cancer is unclear.

Consequently, our study focused on the mechanism of sinomenine in the development and progression of bladder cancer cells. The function of sinomenine was investigated in the proliferation, invasion, migration, and apoptosis of bladder cancer cells, and findings confirmed that sinomenine has a role in bladder cancer by regulating HEIH.

## 2. Materials and Methods

### 2.1. Cell Culture and Treatment

Human bladder cancer cells (T24, 5637, HT-1197, SW780, and TCCSUP) and human normal bladder epithelial cells (SV-HUC-1) were obtained from Bena Culture Collection (Beijing, China). The cell cryopreservation tube was thawed in a water bath at 37°C. 5 ml Roswell Park Memorial Institute 1640 (RPMI-1640) medium containing 10% foetal bovine serum (FBS) was added to the tube and centrifuged at 1000 rpm for 5 min at room temperature. After the supernatant was discarded, 5 ml RPMI-1640 medium was added to prepare cell suspension. The cells were cultured in an incubator with 5% CO_2_ and 37°C. Cell passage was carried out when the cell fusion rate reached 80%.

Sinomenine was purchased from Chengdu Herbpurify Co., Ltd. (Chengdu, China). Sinomenine powder was added to normal saline to configure different concentrations of sinomenine solution (0, 0.25, 0.5, 0.75, and 1 mM). T24 and SW780 cells were treated with sinomenine at different concentrations for 24 h. Also, cells stimulated with dimethyl sulphoxide (DMSO) at the same concentration were used as a control group.

### 2.2. MTT Assay

MTT assay was used to investigate cell viability in T24 and SW780 cells. 100 *μ*L cell suspension was inoculated on 96-well plates with 2000 cells in each well. Cells were cultured in a cell incubator with 5% CO_2_ and 37°C. After culturing for 1, 2, 3, 4, and 5 days, cells were added with 20 *μ*L MTT solution and then cultured for another 4 hours. After added with 150 *μ*L DMSO, the 96-well was shaken on the oscillator for 10 min. The absorbance value at 490 nm was detected with a microplate reader.

### 2.3. Transwell Assay

Transwell assay was carried out to measure cell migration and invasion in bladder cancer cells. 100 cell suspension (1 × 10^5^ cells/ml) was inoculated in a transwell chamber. Different from the migration experiment, matrigel glue was added to the upper chamber for the invasion experiment. After culturing for 48 h, the culture medium was discarded in the well and washed with PBS 3 times. Then, after wiping the upper cells, the chamber was fixed with formaldehyde for 30 mins. Then, cells were stained with 0.1% crystal violet for 30–60 min. Cells were observed and counted in 5 fields randomly by using a microscope.

### 2.4. RT-qPCR Assay

The mRNA expression of HEIH was detected by the RT-qPCR assay. Total RNA was extracted from bladder cancer cells by the TRlzol method. RNA concentration was measured by Nanodrop. Reverse transcription experiments were performed by using PrimeScript RT Reagent Kit with gDNA Eraser. Then, SYBR Premix Ex Taq II was used for the RT-PCR experiment. GAPDH was used as an internal control for HEIH. The primer sequences were as follows: HEIH forward: 5′-ATGCGAGAAGCCATGAGACC-3′, HEIH reverse: 5′-GGAACAGCTTGTGTGACCGA-3′; GAPDH forward: 5′-CTCTGCTCCTCCTGTTCGAC-3′, GAPDH reverse 5′-GACTCCGACCTTCACCTTCC-3′. The relative expressions of HEIH were calculated by the 2^−△△CT^ method.

### 2.5. Western Blotting Assay

The western blotting assay was performed to detect the protein expression of apoptosis-related proteins (Bax, Bcl-2, and caspase-3). Total protein was extracted from the treated cells and quantified by the BCA method. 10 *μ*g total protein was separated by 10% SDS-PAGE protein gel electrophoresis. After membrane transfer, the strips were sealed for 1 h at room temperature. After washing 3 times with TBST, the strips were incubated with the corresponding primary antibody overnight. Then, the strips were placed in the horseradish peroxidase-labeled secondary antibody and incubated. After washing with TBST 3 times, the strips were placed in an ECL kit. Protein expression level = absorbance value of target protein/absorbance value of GAPDH.

### 2.6. Statistical Analysis

GraphPad software was used for the statistical analysis of all data. Data were expressed as mean ± SD. ANOVA and Student's *t*-test were used to analyze the comparison. *p* < 0.05 represented a significant difference.

## 3. Results

### 3.1. HEIH Acted as an Oncogene in Bladder Cancer Cells

**Figure 1:**
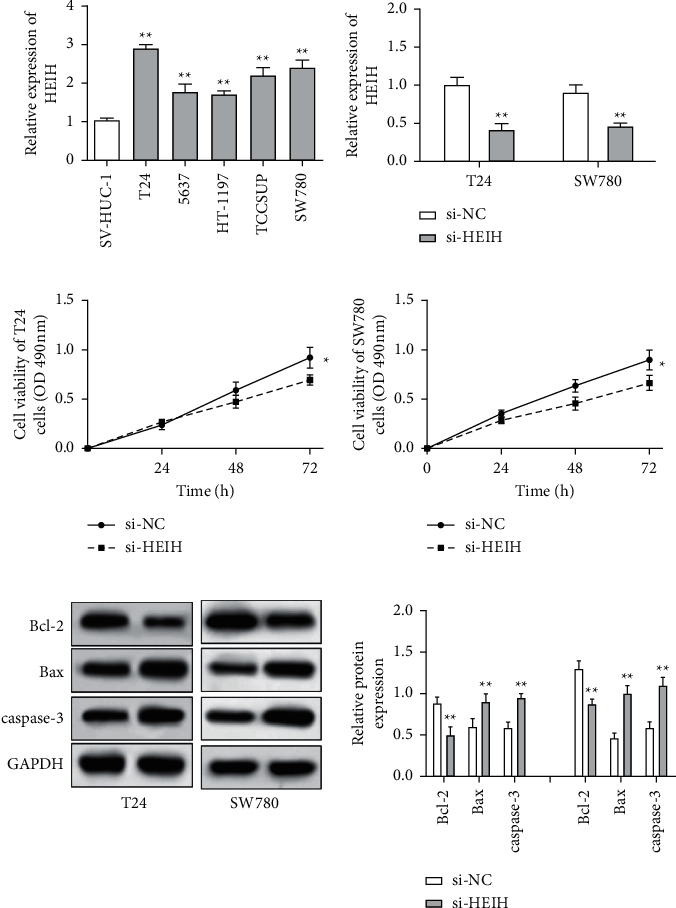
HEIH knockdown inhibited cell viability and accelerated cell apoptosis in bladder cancer cells. (a) HEIH was upregulated in T24, 5637, HT-1197, TCCSUP, and SW780 cells compared with normal bladder epithelial cells (SV-HUC-1). (b) The expression of HEIH was notably reduced in T24 and SW780 cells transfected with HEIH si-RNA. (c, d) HEIH knockdown suppressed cell viability in T24 and SW780 cells. (e) HEIH knockdown accelerated the expression of Bax and caspase-3 but reduced the expression of Bcl-2. ^*∗*^*p* < 0.05; ^*∗∗*^*p* < 0.01.

**Figure 2:**
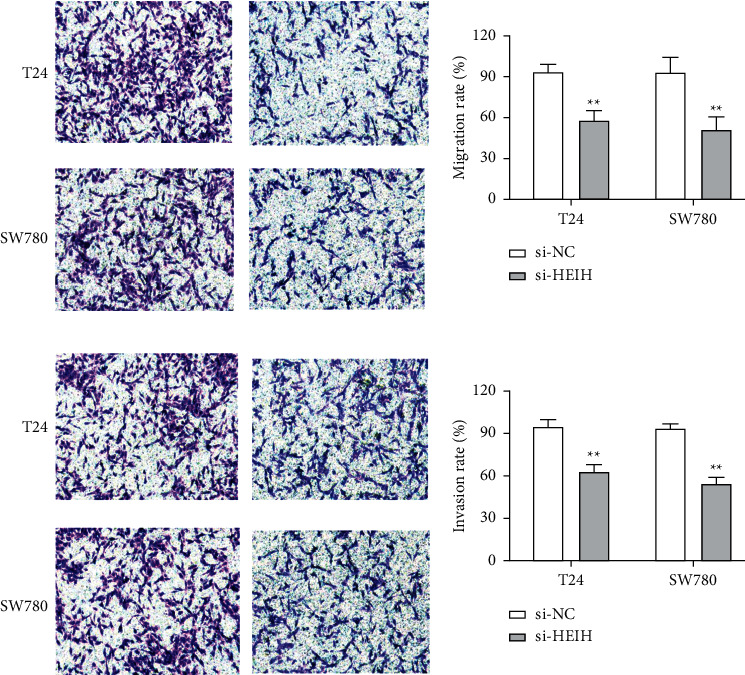
HEIH knockdown suppressed cell migration and invasion in bladder cancer cells. (a) HEIH knockdown significantly suppressed cell migration ability in T24 and SW780 cells. (b) HEIH knockdown significantly suppressed cell invasion ability in T24 and SW780 cells. ^*∗∗*^*p* < 0.01.

In our study, the expression of HEIH was detected by the RT-qPCR assay. Elevation of HEIH was found in T24, 5637, HT-1197, TCCSUP, and SW780 cells compared with normal bladder epithelial cells (SV-HUC-1) (Figure 1(a)). To investigate the function of HEIH in bladder cancer progression, we knocked down the expression of HEIH in T24 and SW780 cells. As shown in Figure 1(b), HEIH expression was downregulated in T24 and SW780 cells' transfection with si-HEIH. Next, the role of HEIH on cell viability, metastasis, and apoptosis in cells was investigated by MTT, transwell, and western blotting assays. Our results showed that HEIH knockdown repressed cell proliferation (Figures 1(c) and 1(d)). In addition, HEIH downregulation facilitated the expression of Bax and caspase-3, but declined the expression of Bcl-2 (Figure 1(e)). The results showed that HEIH knockdown promoted cell apoptosis rate. Next, the inhibiting effect of HEIH silencing was discovered in cell migration and invasion (Figures 2(a) and 2(b)). Taken together, HEIH was observed to be an oncogene in bladder cancer cells.

### 3.2. Sinomenine Suppresses Cell Growth in Bladder Cancer Cells

**Figure 3:**
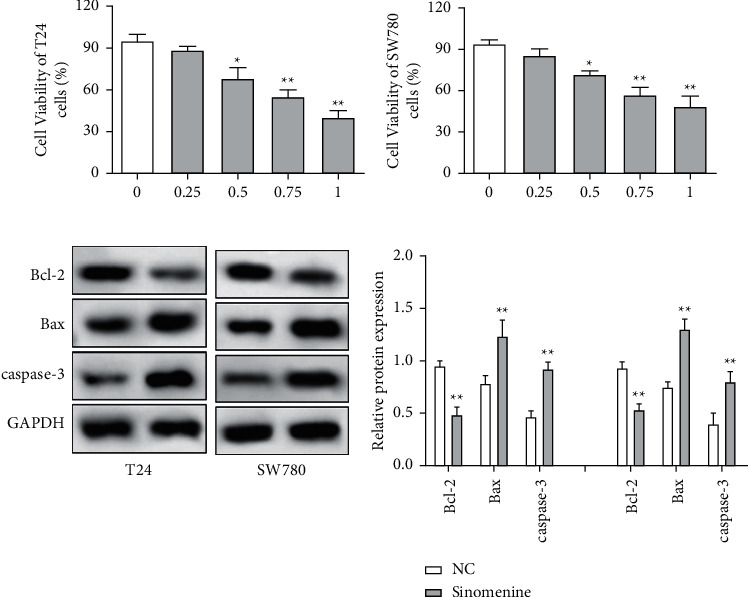
Sinomenine suppressed cell viability and induced cell apoptosis in bladder cancer cells. (a, b) Sinomenine (0, 0.25, 0.5, 0.75, and 1 mM) suppressed T24 and SW780 cell viability in a dose-dependent manner. (c) Sinomenine reduced the expression of Bcl-2 and increased the expression of Bax and caspase-3. ^*∗*^*p* < 0.05; ^*∗∗*^*p* < 0.01.

First, the effects of different doses of sinomenine (0, 0.25, 0.5, 0.75, and 1 mM) on bladder cancer cells were investigated. MTT assay was carried out to detect the cell viability of bladder cancer cells. After treatment with sinomenine for 24 h, cell viability was discovered to be significantly suppressed in a dose-dependent manner (Figures 3(a) and 3(b)). Most importantly, cell viability began to decline considerably at 0.5 mM sinomenine. Hence, 0.5 mM was selected as the appropriate concentration for the subsequent experiments. Next, the expression of cell apoptosis proteins was measured by the western blotting assay. We found that sinomenine accelerated the expression of Bax and caspase-3, but decreased the expression of Bcl-2 (Figure 3(c)). These results imparted that sinomenine restrained cell viability and induced cell apoptosis in bladder cancer cells.

### 3.3. Sinomenine Inhibited Cell Migration and Invasion in Bladder Cancer Cells

**Figure 4:**
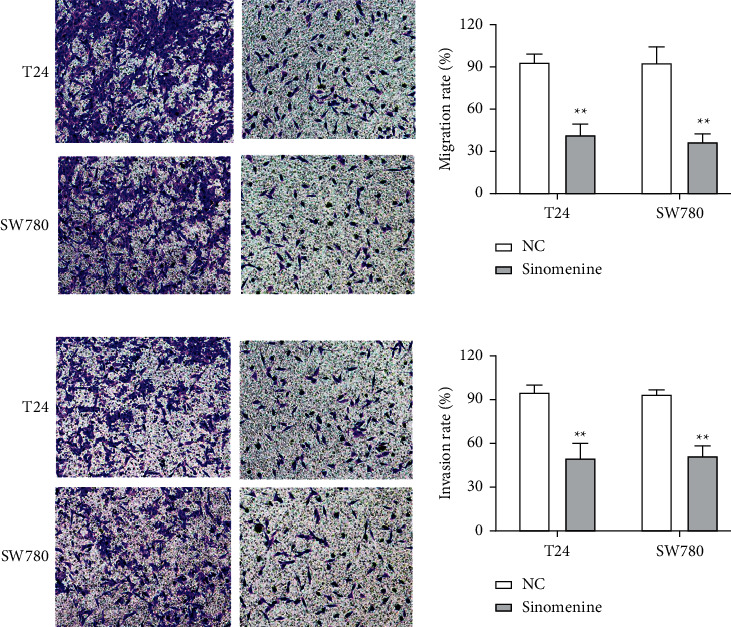
Sinomenine suppressed cell migration and invasion in bladder cancer cells. (a) Sinomenine significantly suppressed cell migration ability in T24 and SW780 cells. (b) Sinomenine notably inhibited cell invasion ability in T24 and SW780 cells. ^*∗∗*^*p* < 0.01.

Transwell assay was used to assess the effect of sinomenine on the cellular motility of bladder cancer cells. We found that cell migration capacity was notably reduced in T24 and SW780 cells treated with sinomenine (Figure 4(a)). Likewise, similar results were discovered in the cell invasion experiment. Sinomenine suppressed cell invasion capacity in T24 and SW780 cells (Figure 4(b)). All the observations declared that sinomenine could block cell migration and invasion in bladder cancer.

### 3.4. Sinomenine Acted the Antigrowth Effect in Bladder Cancer Cells by Inhibiting HEIH Expression

**Figure 5:**
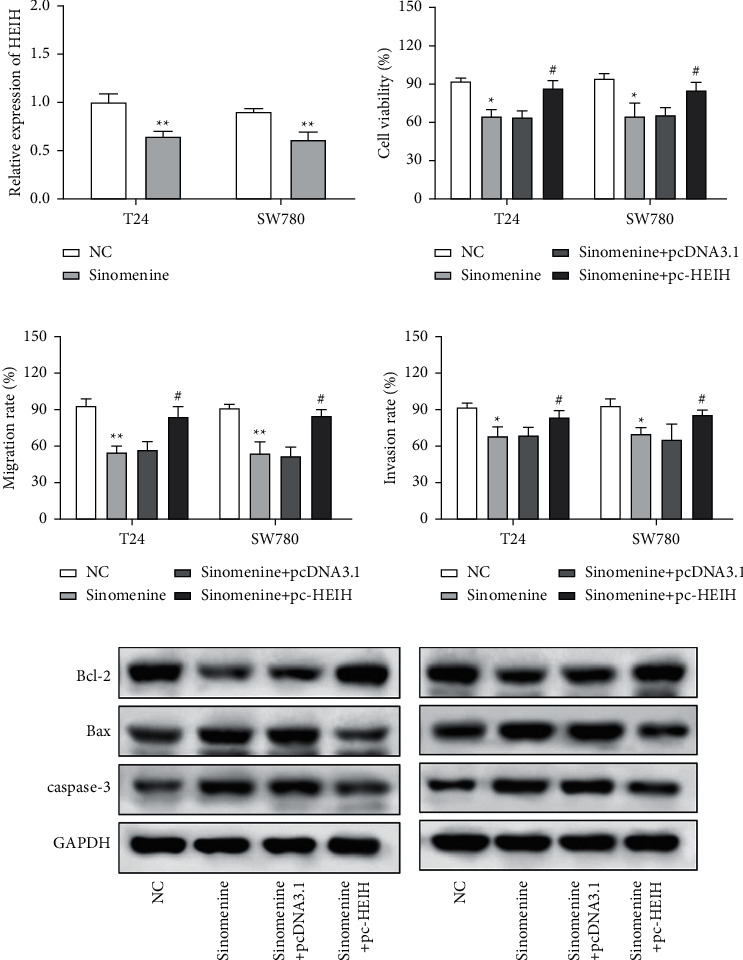
Sinomenine acted the antigrowth effect in bladder cancer cells by inhibiting HEIH expression. (a) Sinomenine suppressed the expression of HEIH. (b–e) ^*∗*^*p* < 0.05 and ^*∗∗*^*p* < 0.01 compared with the control group; ^#^*p* < 0.05 compared with the sinomenine + pcDNA3.1 group.

Next, the expression of HEIH in bladder cancer cells stimulated with sinomenine was investigated. We noticed that HEIH was lower in bladder cancer cells after manipulation with sinomenine (Figure 5(a)). To investigate how sinomenine regulated HEIH in the progression of bladder cancer, T24 and SW780 cells transfected with pc-HEIH were treated with sinomenine. The data showed that HEIH reversed the suppression of cell proliferation induced by sinomenine (Figure 5(b)). As we expected, the sinomenine-induced decline in cell migration was restored by HEIH overexpression (Figure 5(c)). Similarly, the suppression of cell invasion induced by sinomenine was restored by HEIH overexpression (Figure 5(d)). Besides this, HEIH reduced cell apoptosis induced by sinomenine (Figure 5(e)). Therefore, sinomenine was confirmed to suppress the progression of bladder cancer by inhibiting HEIH expression.

## 4. Discussion

In China, TCM is used in almost every aspect of cancer treatment. Rational application of TCM in tumor treatment can potentiate the curative effects, effectively control tumor recurrence and metastasis, and prolong the survival time [17, 18]. The function of TCM in the procession of bladder cancer has been widely studied in recent years. For example, orientin repressed bladder cancer cell growth and facilitated cell apoptosis by downregulating NF-kappaB and Hedgehog pathway [19]. Curcumin inhibited bladder cancer cell growth through multiple signaling pathways [20, 21]. In our experiment, we investigated the role of sinomenine on bladder cancer cells. Sinomenine was discovered to suppress cell viability, migration, and invasion and facilitate cell apoptosis in T24 and SW780 cells. Besides this, our findings indicated that sinomenine possesses antitumor effects by suppressing HEIH expression in bladder cancer.

Sinomenine is an alkaloid monomer extracted from *Sinomenium acutum*. It has unique pharmacological properties and can be used in the treatment of inflammatory and autoimmune diseases [22]. Sinomenine has been widely reported in the treatment of rheumatoid arthritis and neuralgia [9, 23]. In addition, the antitumor effects of sinomenine have been found in various cancers. Shen et al. reported that sinomenine restrained migration and invasion of lung cancer cells by repressing miR-211 and MMPs [24]. Moreover, sinomenine enhanced renal carcinoma cell apoptosis by promoting autophagy and suppressing the PI3K/AKT/mTOR pathway [25]. Consistent with our results, sinomenine was proved to block cell proliferation, migration, and invasion but induce cell apoptosis in prostate cancer [26]. Furthermore, Yuan et al. documented that sinomenine blocked cell growth and evoked cell apoptosis in gastric cancer cells, which is comparable to our findings [27]. Additionally, sinomenine inhibited glioma cell growth and promoted *G*0/*G*1 cell cycle arrest by promoting p53 and downregulating SIRT1 expression [28]. In consistency with previous studies, we confirmed that sinomenine could effectively repress the progression of bladder cancer.

LncRNA-HEIH has been reported to be carcinogenic in various cancers. In this experiment, we found that HEIH was upregulated in bladder cancer cell lines (T24, 5637, HT-1197, TCCSUP, and SW780). Additionally, HEIH knockdown was confirmed to block cell growth and motility, but evoke cell apoptosis in bladder cancer cells. Consistent with our results, Gao's study has shown that HEIH was overexpressed in retinoblastoma, and HEIH knockdown remarkably suppressed the viability, migration, and invasion of retinoblastoma cells [29]. Moreover, in ovarian cancer, HEIH facilitated cell progression and blocked cell senescence by regulating miR-3619-5p and CTTNBP2 [30]. Furthermore, HEIH silencing was observed to downregulate Bcl-2, cyclin D1, vimentin, MMP-2, and MMP-8 and upregulate Bax, cleaved caspase-3, and p53 [31]. Similar to previous results, we found that HEIH knockdown reduced the expression of Bcl-2 but accelerated the expression of Bax and cleaved caspase-3. Moreover, we investigated the relationship between sinomenine and HEIH in bladder cancer progression. Our results indicated that HEIH reversed the suppression of cell progression induced by sinomenine. However, the effect of sinomenine on bladder cancer was only in vitro, so more in vivo experiments should be carried out in the future to clarify its antitumor mechanism.

## 5. Conclusion

In conclusion, we reported that sinomenine blocked cell viability, migration, and invasion and induced cell apoptosis in bladder cancer cells by suppressing HEIH expression. Therefore, we confirmed the antitumor effect of sinomenine, providing a new idea and potential approach for treating bladder cancer.

## Data Availability

The data used to support the findings of this study are available from the corresponding author upon request.
